# Tetrandrine inhibits colon carcinoma HT-29 cells growth via the Bcl-2/Caspase 3/PARP pathway and G1/S phase

**DOI:** 10.1042/BSR20182109

**Published:** 2019-05-14

**Authors:** JiaNan Li, QiuHong Wang, ZhiBin Wang, Na Cui, BingYou Yang, WenYing Niu, HaiXue Kuang

**Affiliations:** 1 Key Laboratory of Chinese Materia Medica (Ministry of Education), Heilongjiang University of Chinese Medicine, Harbin, China; 2 Science of Processing Chinese Materia Medica, College of Pharmacy, Guangdong Pharmaceutical University, Guangzhou, China; 3 Experimental training center, Heilongjiang University of Chinese Medicine, Harbin, China

**Keywords:** bioinformatics, colon cancer, cell apoptosis, cell cycle, molecular docking, Tetrandrine

## Abstract

Tetrandrine (Tet) *bisbenzylisoquinoline* alkaloids isolated from *Stephania tetrandra* and other related species of *Menispermaceae.* It has been demonstrated to have positive therapeutic effects on cardiovascular disease, hypertension, silicosis, autoimmune diseases. In recent years, some reports have shown that Tet has anticancer activity in human cancers. To explore the pharmacological activity and mechanism of Tet on colon cancer and its unique advantages as a natural product. In the present study, analyses of the cell cycle, apoptosis, targets prediction, molecular docking, and alterations in protein levels were performed to elucidate how Tet functions in colon cancer. We found that Tet robustly induced arrest at the G1 phase in colon cancer cell line HT-29. It induced HT-29 cell apoptosis in a dose-dependent manner. Similarly, analysis of protein expression levels in HT-29 cells showed down-regulation of Bcl-2, pro-caspase 3, pro-caspase 8, PARP, cyclin D1 (CCND1), cyclin-dependent kinase 4 (CDK 4), and up-regulation of Bax, active caspase 3, and active caspase 8. These results indicate that Tet induces apoptosis of colon cancer cells through the mitochondrial pathway and caspase family pathway. Molecular docking showed interaction effects and binding energy. Comparing with the CDK4 inhibitors ribociclib and palbociclib, the docking energy is similar to the docked amino acid residues. Therefore, we conclude that Tet and the CCND1/CDK4 compound could form hydrogen bonds and a stable compound structure, which can inhibit colon cancer cells proliferation by regulating CCND1/CDK4 compound and its downstream proteins phosphorylated Rb (p-Rb). In summary, Tet may be a potential drug for colon cancer therapy.

## Introduction

Tetrandrine (Tet), *bisbenzylisoquinoline* alkaloids isolated from *Stephania tetrandra* and other related species of *Menispermaceae*, is considered to have significant biological activities. As is well-known, Tet as a calcium channel blocker has been verified in clinical trials and found to effectively against silicosis, hypertension, inflammation, and cancer without any toxicity [1]. There are some studies showing that Tet is a robust anticancer drug based on *in vitro* and *in vivo* against a wide range of cancers of the breast, liver, pancreatic, leukemia, lung, prostate, etc. [2–4]. Much evidence reveals that Tet is a potential candidate drug to treat cancer. Such as, Tet exhibits a reversal of drug resistance by modulating P-glycoprotein (P-gp) expression levels in different tumors, which are an added advantage of this natural compared with other chemotherapy drugs [5,6]. Moreover, paclitaxel and camptothecin have been widely studied over decades for treating cancer. Natural products play an essential role in human diseases.

Colon carcinoma cell is the most common form of colorectal cancer and represents a significant health issue as it is the most common gastrointestinal tract cancer worldwide with over 1.2 million new diagnoses each year [7], which is the third most commonly diagnosed cancer in males and the second in females [8]. Colon cancer is generally diagnosed at a later stage of cancer development. Understanding the apoptosis pathways and their corresponding inhibitors enables us to formulate strategies for cancer therapy. The p53 tumor suppressor gene is highly involved in cell cycle regulation, DNA repair, and programmed cell death [9]. It has been shown in several cell types that wild-type (wt) p53 is required for the apoptosis cell death as induced by a variety of anticancer drugs. The mechanisms include transcriptional activation of the apoptosis Bcl-2 family member Bax and caspase family signaling pathway [9]. HT-29 is one of colon cancer cell lines characterized by invasion and migration. Besides, it is a mutant cell line with mutation site information (G→A, Arg-273→His). Mutation of the p53 gene is one of the most frequent genetic changes in the development of human colorectal cancer [10]. However, the wt p53 gene usually mediates apoptosis pathways such as the mitochondrial apoptosis pathway Bcl-2 family and the apoptosis caspase signaling pathway. In the absence of the functional p53 protein, apoptosis can still occur in HT-29 cells, suggesting that apoptosis does not requires the activation of the p53 gene. Similarly, the p21 gene is regulated by the wt p53, yet the cell cycle G1 arrest still occurs when the p21 gene is inactivated. Therefore, this research founded that Tet could induce apoptosis and cell cycle arrest through the p53-independent pathway.

## Materials and methods

### Materials

Tet (purity 99.1%) was purchased from Alphabio Biotechnology Co. Ltd (Tianjin, China). The HT-29 cell line was obtained from Cell Bank of Shanghai Institutes for Biological Sciences, Chinese Academy of Sciences (Shanghai, China). DMEM was purchased from Corning Cellgro Inc. (Herndon, VA, U.S.A.) and the fetal bovine serum (FBS) was obtained from Biological Industries Technologies (Kibbutz Beit Haemek, Israel). DMSO and MTT were acquired from Beyotime Biotechnology Co., Ltd. (Shanghai, China). Trypsin-EDTA solution, penicillin-streptomycin solution, mitochondrial membrane potential (MMP) assay kit with JC-1, caspase 3, 8 activity assay kit, and propidium iodide (PI)/RNase staining solution were obtained from Beyotime Biotechnology Co., Ltd. (Shanghai, China). FITC goat anti-rabbit IgG and Annexin V-FITC Apoptosis Detection Kit were acquired from Tianjin Sungene Biotech Co. Ltd. (Tianjin, China). Anti-Bax, anti-Bcl-2, anti-caspase 3, anti-caspase 8, anti-PARP, anti-cyclin D1 (anti-CCND1), anti-cyclin-dependent kinase 4 (anti-CDK4), anti-phosphorylated Rb (anti-p-Rb) (Ser780), and β-actin antibodies were purchased from Bioss Biotechnology Co. Ltd. (Beijing, China).

### Cell culture

HT-29 cells were cultured in DMEM supplemented with 10% FBS, and 1% penicillin-streptomycin. All cells were incubated at 37°C in an atmosphere of 5% CO_2_. After cells were grown to the logarithmic growth phase, they were digested with 0.25% trypsin.

### Cell viability assay

The MTT assay determined HT-29 cells in viability following treatment with Tet. Cells were seeded at a density of 5 × 10^3^ cells/well and allowed to attach for 24 and 48 h in 96-well plates. According to the experimental design, different concentrations of Tet (5, 10, 20, 40, and 80 μM) were added 96-well plates for 24 and 48 h. After that, the 10 μl volume of 5 mg/ml MTT was added to each well and incubated for another 4 h at 37°C. The medium was removed, 150 μl DMSO was added to each well, and the absorbance was measured at 570 nm with a multi-detection microplate reader (PerkinElmer, VICTOR X 3, U.S.A.).

### Detection of apoptosis

Cell apoptosis was determined using the Annexin V-FITC apoptosis detection kit. Briefly, HT-29 cells (2 × 10^6^ cells/ml) were seeded in six-well plates and cultured for 24 h with different concentrations in DMEM. After 24 h, all adherent cells were collected with 0.25% (w/v) trypsin without EDTA, including floating cells in the medium. Annexin V-FITC and PI were used to label cells according to the manufacturer’s instructions. The double-stained cells were subsequently analyzed by a BD Accuri®C6 flow cytometer (BD, U.S.A.). Cells stained with both Annexin V-FITC and PI were considered to be late apoptosis, and the cells stained only with Annexin V-FITC was considered to be early apoptosis. At least 10000 cells were counted each time.

### MMP assay

MMP was evaluated by cationic dye JC-1. In normal cells, JC-1 aggregates in mitochondria, fluorescence red. In apoptosis cells, JC-1 accrues in the cytosol, as a green fluorescence monomer. HT-29 cells were harvested by trypsin. After two PBS washing, cells were incubated with JC-1 10 mg/ml for 20 min at 37°C in the dark. Cells were observed by the inverted microscope (Olympus, Japan).

We also observed the nuclear morphology of HT-29 cells subjected to Hoechst 33342 fluorescent staining after treatment for 24 h with different concentrations Tet; the HT-29 cells were washed with PBS and fixed with methanol:acetic acid (3:1) for 15 min at room temperature. The cells were washed with PBS and stained with 5 μg/ml Hoechst 33342 for 10 min. Alterations in the morphology of the nuclear were observed using fluorescence microscopy (Leica, Germany).

### Flow cytometry analysis of DNA content

HT-29 cells were seeded into an in six-well plates with different concentrations of Tet for 24 h. The cells were digested with 0.25% trypsin and re-suspended in 1 ml 1× PBS before centrifugation at 1000×***g*** for 5 min. They were then fixed in 70% ethanol at 4°C for 2 h. Before analysis, cells were washed twice in PBS and stained with PBS containing 25 μl PI (20×) and 10 μl RNase (50×) for 30 min at 37°C. Analyses were performed with a BD Accuri®C6 flow cytometer. Cell cycle distribution was calculated on DNA plots by Mod Fit LT software (Verity Software House, Inc., Topsham, ME).

### Western blot analysis

HT-29 cells were cultured in 60 mm dishes. After HT-29 cells were treated with various concentrations of Tet (0, 10, 20, and 30 μM), then lysed in cell buffer, and determined using the BCA assay kit. Then, an equal amount of protein buffer was separated by 4–20% BeyoGel™ Plus Precast PAGE Gel (Beyotime, China), and was transferred to 0.22 μm NC membranes. The membranes were blocked with 5% (w/v) non-fat milk in PBST for 2 h. All the primary antibodies were incubated at 4°C overnight. After being washed with PBST, the membranes were incubated with the secondary anti-rabbit antibody at room temperature for 1 h. After being washed with PBST, bands were scanned by Odyssey® CLx Infrared Imaging System (LI-COR, U.S.A.).

### Detection of caspase 3 and caspase 8 activity

An additional test was performed to assess HT-29 cells apoptosis with higher specificity. HT-29 cells caspase 3 and caspase 8 activity was determined via caspase 3 and caspase 8 activity assay kit. In brief, HT-29 cells were seeded into six-well plates with different concentrations of Tet for 24 h. The sample was washed with PBS for three times and added with cell lysis buffer, and then re-centrifuged 14000×***g*** for 15 min. Supernatant samples were used to measure the caspase 3 and caspase 8 activity assay, and protein concentrations were measured by the Bradford method. Then, 50 μl of supernatant with 10 μl (2 nM) of caspase 3 and caspase 8 substrate acetyl-Asp-Glu-Val-Asp p-nitroanilide (Ac-DEVD-pNA) and acetyl-Ile-Glu-Thr-Asp (Ac-ITED-pNA) was incubated at 37°C for 2 h. Finally, the absorbance of yellow pNA was calculated with a spectrometer at 405 nm.

### Molecular docking

TCMSP is a database of systems pharmacology for drug discovery from herbal medicines. The particular strengths of TCMSP are the information of a large number of herbal entries, and the ability to identify drug–target networks and drug–disease networks, which will help to reveal the mechanisms of action of Chinese herbs. The targets in the TCMSP were obtained from two sources; one is the experimentally validated drug–target, the other compounds without validated targets. The SysDT model was used to predict the potential targets of a compound. SysDT shows the impressive performance of prediction for drug–target interactions, with a concordance of 82.83%, a sensitivity of 81.33%, and a specificity of 93.62%, respectively [11].

The molecular docking is used to determine the binding mode of small molecules in the complex to protein targets, and the critical interactions between them are studied, which provides a theoretical basis for determining the mechanism of action of drugs. The spatial structure of Tet was downloaded from the ChemSpider database with MOL format, supported by chem3D Pro 14.0, then calculated minimum energy, and saved as PDB format. The protein structures of targets were downloaded from RCBS Protein Data Bank. Deleting water molecules and adding polar hydrogen atoms, then generatingtransforming growth factor β-1 a PDBQT file, respectively. Molecular docking was done by a program named AutoDock Vina, which an open-source and high accuracy program. Docking results were plotted and displayed using Pymol software.

### Statistical analysis

All statistical tests were performed with GraphPad Prism software, version 5.0 (GraphPad Software, San Diego, CA). Results are expressed as the mean ± SD (standard deviation). The difference between means was tested using the Student’s *t* test, and statistical significance was assumed for *P*-values <0.05. All data were performed at least three times.

## Results

### Effects of Tet on cell viability in HT-29 colon cancer cells

**Figure 1:**
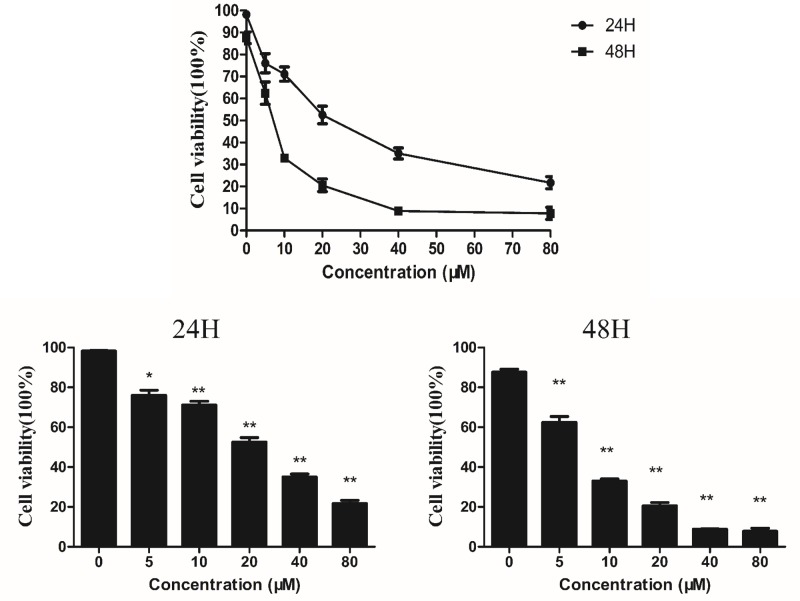
Assessment of the cell viability (MTT assay) of HT-29 cells following treatment with Tet at the indicated concentrations

Figure 1 shows the cell viability of HT-29 cells following treatment with different concentrations of Tet for 24 and 48 h. The viability of cells significantly decreased when Tet concentration was increased. The average inhibition rate at 5 μM was 23.94 and 37.54%; the average inhibition rate was 28.87 and 67.03% at 10 μM; the average inhibition rate was 47.49 and 79.42% at 20 μM; the average inhibition rate was 64.96 and 91.14% at 40 μM, and 78.29 and 92.23% at 80 μM. The IC_50_ of cells after 24 and 48 h of incubation were found to be 22.98 and 6.87 µM, respectively. After 48 h of incubation, the cell viability was obviously reduced.

### Tet-induced HT-29 cancer cells apoptosis

To show that Tet induces apoptosis, Annexin V-FITC/PI staining assays were performed. Annexin V is a member of the Annexin family of intracellular proteins that bind to phosphatidylserine (PS) in a calcium-dependent manner. PS is usually only found in the intracellular leaflet of the plasma membrane in healthy cells, but during early apoptosis, membrane asymmetry is lost, and PS translocates to the external leaflet. Fluorochrome-labeled Annexin V can be used to target and identify apoptosis cells accurately. PI can stain necrotic cells or cells that lose cell membrane integrity in the late stage of apoptosis, showing red fluorescence.

**Figure 2:**
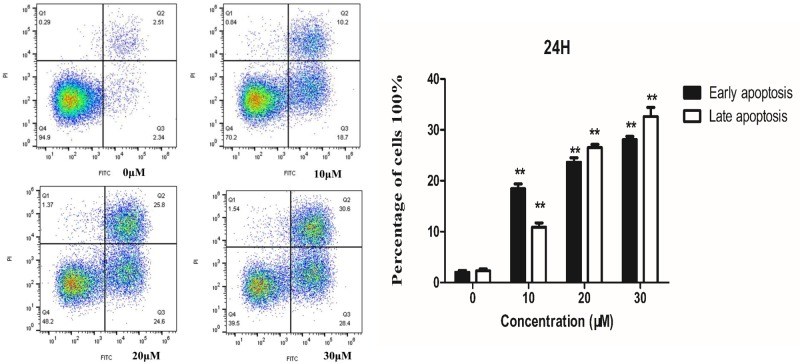
Flow cytometry analysis of HT-29 cells treated with Tet for 24h Left: Detection of apoptosis by Annexin V-FITC/PI staining. Right: Quantification of apoptotic cells.

For necrotic cells, since the integrity of the cell membrane has been lost, Annexin V-FITC can enter the cytoplasm and bind to PS located inside the cell membrane; thereby, causing necrotic cells to exhibit green fluorescence. As shown in Figure 2, each area can be divided into four parts. The bottom left quadrant contains viable cells, which exclude PI and is negative for Annexin V binding. The bottom right quadrant contains early apoptosis cells, which are positive for Annexin V binding and exclude PI. The top right quadrant contains late apoptosis cells or necrotic cells, which are positive for PI and Annexin V. The percentage of apoptotic cells was increased after Tet treatment, which Annexin V positive cells increased in dose-dependent manners.

### Tet decreased MMP (ΔΨm) in colon cancer and morphological observation

**Figure 3:**
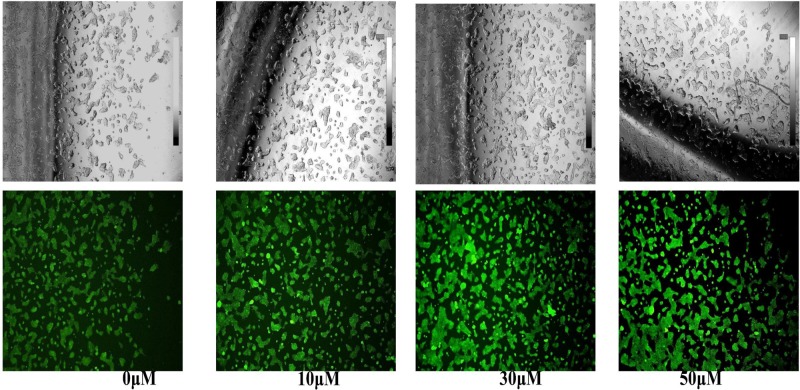
After treatment with a high concentration of Tet for 24 h, the morphology of the cells changed significantly, and the MMP (ΔΨm) decreased

**Figure 4:**
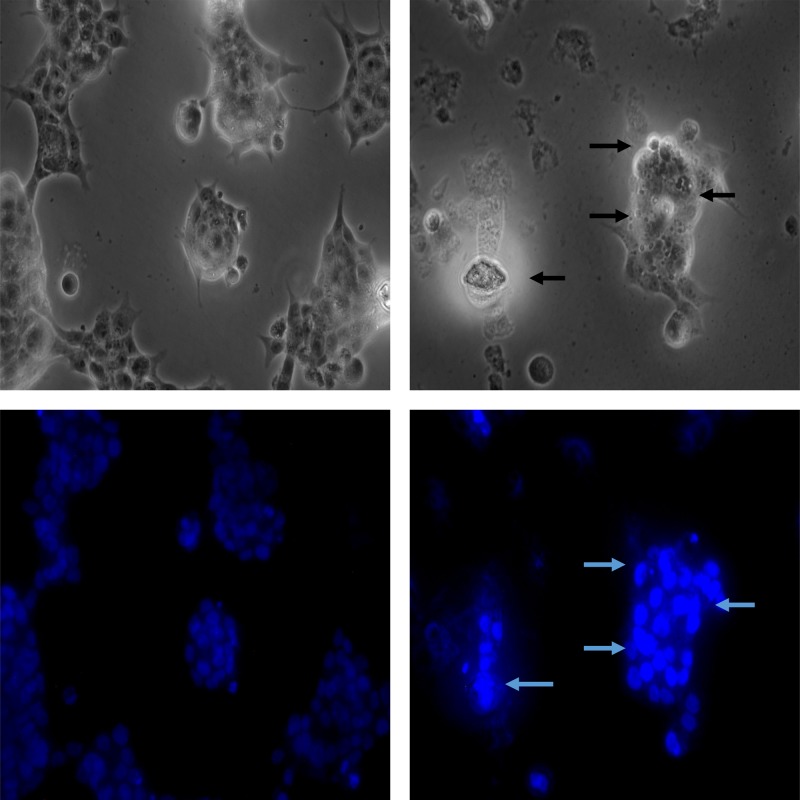
Tet induces apoptosis in HT-29 cells Cells were treated with 30 μM Tet for 24 h and then stained with Hoechst 33342. Left: control; Right: Tet.

The decreased MMP is an iconic event in the early stages of apoptosis. The decrease of cell membrane potential can be easily detected by the transition of red fluorescence to green fluorescence of JC-1, and the change from red fluorescence to green fluorescence of JC-1 can also be used as a detection index for early apoptosis. Comparing with the control and low dose (0 and 10 μM) groups, cells treated with Tet at high doses (30 and 50 μM) showed increased proportion of green fluorescence, which indicates a remarkable decrease in ΔΨm (Figure 3). Besides, to test whether Tet could induce colon cancer cell death, we used Hoechst staining to observe the morphological alterations of nuclei. In the untreated HT-29 cells, the observed nuclei were a weak homogeneous blue, whereas bright chromatin condensation and nuclear fragmentation were observed in the group treated with Tet. The number of apoptotic nuclei containing condensed chromatin increased significantly (Figure 4) suggesting that Tet may induce colon cancer cell apoptosis.

### Tet-induced G1 phase cell cycle arrest

**Figure 5:**
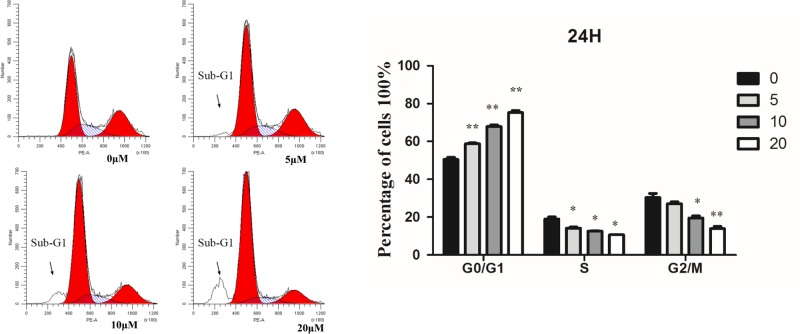
Flow cytometry analysis of HT-29 cells treated with Tet for 24 h HT-29 cells were incubated with 0, 5, 10, and 20 µM of Tet for 24 h. The right graphs showed the cell cycle analysis (%) of HT-29 cells. *P<0.05, **P<0.01 vs control group.

HT-29 cells were treated with different concentrations of Tet for 24 h, and their cyclical changes were detected by flow cytometry. As showed in Figure 5, at 5 μM the G1 phase of cells increased from 50.42 to 58.74%, while the proportion of S phase and G2/M phase decreased significantly. With the increase in concentration, the percentage of G1 increased to 75.85%, and the ratio of S and G2/M decreased gradually compared with other groups. These results indicated that Tet could significantly induce HT-29 cell arrest. During the process of apoptosis, endonuclease degradation in the nucleus of the molecule led to DNA fragmentation, and decreased nuclear content. Flow cytometry analysis revealed a sub-diploid peak (sub-G1) in front of the G0/G1 peak of normal diploid cells, which represents apoptosis cells.

### Effect of proteasome inhibitor on the induction of cell cycle arrest

To explore whether the proteasome is involved in the down-regulation of CCND1, the effects of Tet were studied in the presence of the proteasome inhibitor MG132. Because MG132 itself is cytotoxic, exponentially growing HT29 cells were used in the following experiments. As shown in Figure 7, CCND1 started to decrease after 24 h of exposure to Tet alone. Pretreatment with MG132 blocked the Tet-induced reduction of CCND1, suggesting the involvement of the proteasome in the degradation of CCND1/CDK6 and CDK4 complex. These results show that CCND1 is degraded by an ubiquitin-mediated pathway after exposure to Tet. Proteasome pathway activation may be involved in the induction of cell cycle arrest.

### Effects of tet regulated caspase 3, caspase 8, PARP, Bax and Bcl-2 levels in HT-29 cells

To further elucidate how Tet induced colon cancer cell apoptosis, we measured the expression of apoptosis-associated genes. We observed decreased levels of pro-caspase 3, pro-caspase 8, and full-length-PARP, demonstrating the induction of apoptosis by Tet in colon cancer cells. Interestingly, we found that Tet decreased the anti-apoptotic protein Bcl-2 and increased the pro-apoptotic protein Bax. Considering that these two proteins are closely associated with mitochondria, it is possible that Tet induced apoptosis through mitochondrial disruption by changing the levels of the apoptosis-associated proteins Bcl-2 and Bax (Figures 8 and 9A–C). Figure 9D showed the activity of caspase 3, 8 in HT-29 cells after treatment of Tet. According to the kit instructions, one unit is the amount of enzyme that will cleave 1.0 nmol of the colorimetric substrate Ac-DEVD-pNA per hour at 37°C under saturated substrate concentrations. We normalized each group by protein concentration and then defined its activity by per mg of protein.

### Target verification and molecular docking

**Figure 6:**
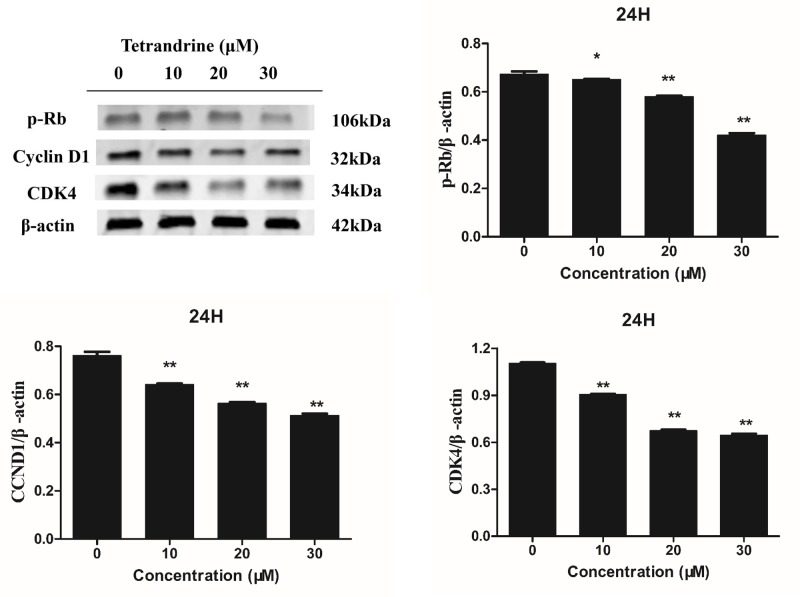
Effects of Tet on the levels of CCND1, CDK-4, and P-Rb proteins in HT-29 colon cancer cell β-actin was used as an internal control and quantification of protein expression relative to the internal control β-actin. *P<0.05, **P<0.01 vs control group.

As shown in Table 1, we obtained potential targets for Tet from the TCMSP bioinformatics database. Interestingly, the targets we got from the database are mostly related to the cell cycle G1 (Kyoto Encyclopedia of Genes and Genomes pathway: map04110, cell cycle), such as transcription factor E2F1 (E2F1), transforming growth factor β-1 (TGFB1), CDK4, CCND1, and cyclin-dependent kinase inhibitor 1 (CDKN1A). As shown in Figure 6, CCND1, CDK, and p-Rb protein expression levels are decreased. Due to the inactivation of wt p53, the level of p21 protein is not detected in the mutant HT-29 cell line. The downstream gene of p21 decreased in a dose-dependent manner under the action of Tet. These results indicate that Tet may act as a CCND1/CDK4 complex inhibitor, and inhibit its downstream phosphorylated Rb protein. The crystal structure of CDK4 in complex with a D-type cyclin (PDB ID: 2W96) was downloaded from RCSB PDB. We separated the ligand (GOL) from the complex and re-docked it to derive the RMSD value to assess the effectiveness of the docking; the RMSD value was 1.34 A, and its docking method was reliable. Figure 10 shows the docking of the receptor and the ligand, where the active pocket center (17.069, 9.861, 59.183), number of points (42, 42, 44), spacing: 0.403 and 50 runs. From the analysis of ligand and co-crystal structure, the ligand can form hydrogen bonds with ARG-87, LEU-148, ASN-151, THR-37 amino acid residue. In addition, we analyzed the CDK inhibitor (CKI) ribociclib (Figure 10B) and palbociclib (Figure 10C) binding complex. Above all, we found that its binding mode is similar to the binding of GOL ligand, and more amino acid residues to make the binding more stable. Finally, we docked Tet with the complex. We found that Tet can form hydrogen bonds with ARG-87, ASN-151, and ALA-39 amino acid residues, similar to previous ligands and inhibitors. Regarding energy, Tet, ribociclib, palbociclib, and GOL are −5.16 kcal/mol, −6.29 kcal/mol, −6.95 kcal/mol, and −2.66 kcal/mol. Its binding energy is lower than that of the ligand and similar to the inhibitor. These results suggest that Tet can be similar to the binding mode of ribociclib, palbociclib to CCND1/CDK4 complex, and play a regulatory role.

**Figure 7:**
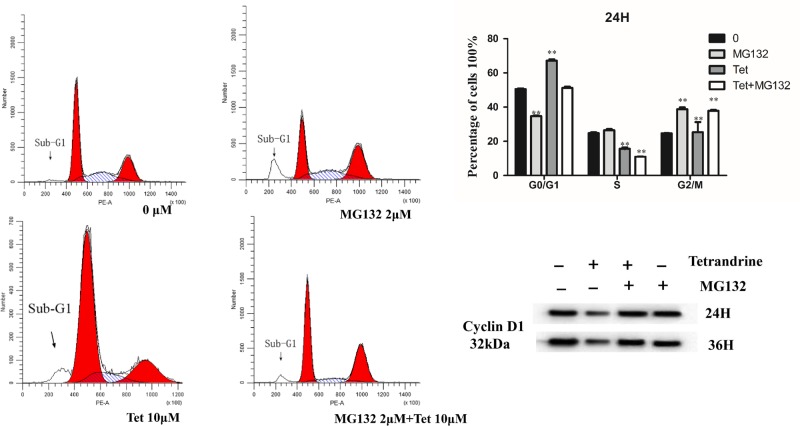
Flow cytometry analysis of HT-29 cells treated with Tet and MG132 for 24 h HT-29 cells were incubated with 0, 2 µM MG132, 10 µM Tet, 2 µM MG132, and 10 µM Tet for 24 h. The right graphs showed the cell cycle analysis (%) of HT-29 cells and effects of Tet the levels of CCND1 proteins in HT-29 colon cancer cell after treated with Tet and MG132 for 24 and 36 h. *P<0.05, **P<0.01 vs control group.

**Figure 8:**
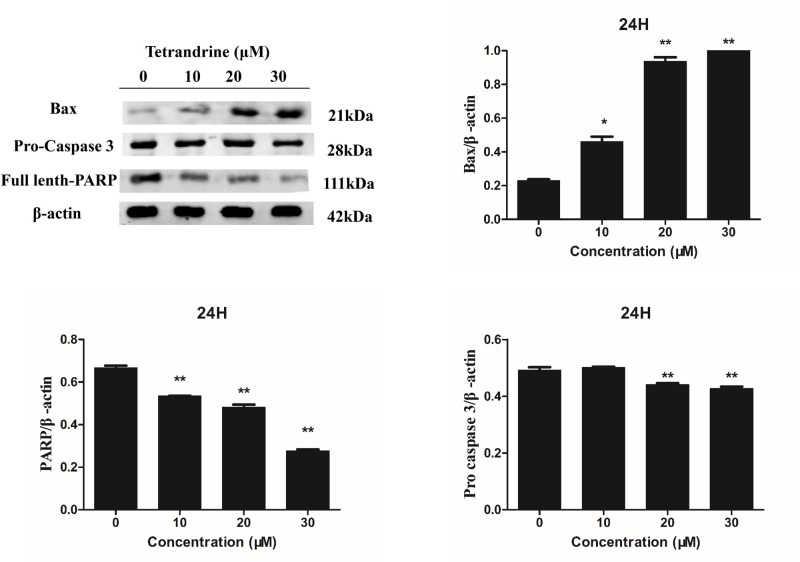
Effects of Tet the levels of Pro-caspase 3, Bax, and PARP proteins in HT-29 colon cancer cell β-actin was used as an internal control and quantification of protein expression relative to the internal control β-actin.

**Figure 9:**
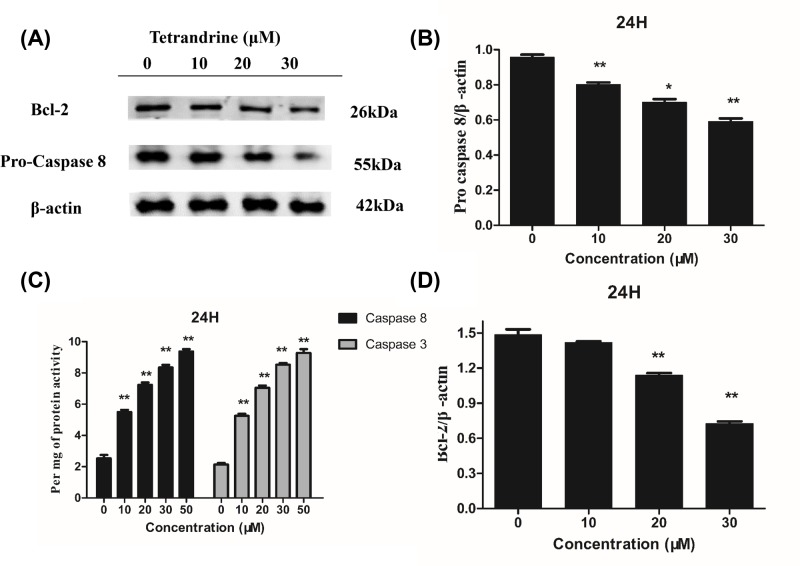
Effects of Tet the levels of Pro-caspase 8, Bcl-2 proteins in HT-29 colon cancer cell β-actin was used as an internal control. (**B,D**) Quantification of protein expression relative to the internal control β-actin. (**C**) Active-caspase 3, 8 activity. *P<0.05, **P<0.01 vs control group.

**Figure 10:**
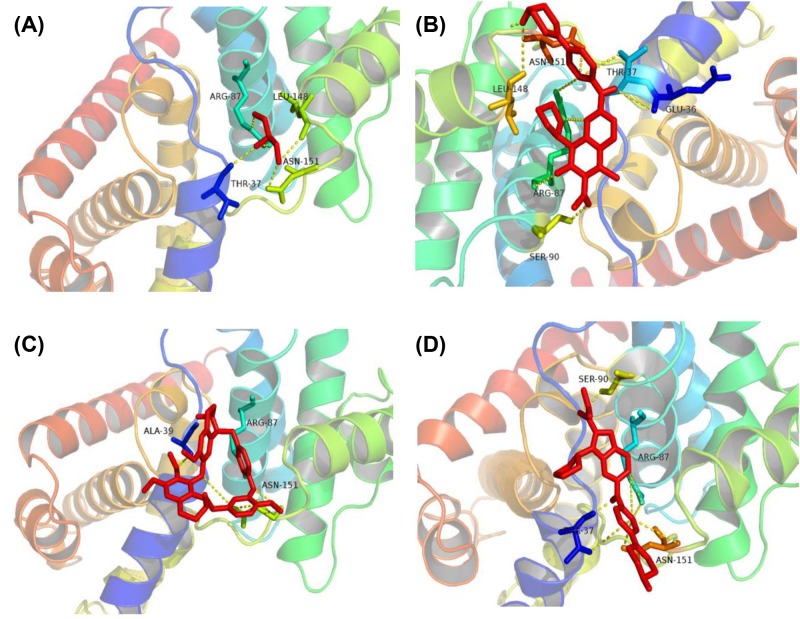
Molecular docking site (**A**) The docked pose of GOL into the CCND1/CDK4 complex binding site. (**B**) The docked pose of ribociclib into the CCND1/CDK4 complex binding site. (**C**) The docked pose of palbociclib into the CCND1/CDK4 complex binding site. (**D**) The docked pose of Tet into the CCND1/CDK4 complex binding site.

**Table 1:** The potential targets information on cancer

PDB ID	Target name	Uniprot ID	Gene	Resource
2AZE	E2F1	Q01094	E2F1	TCMSP
1FOS	Proto-oncogene c-Fos	P01100	FOS	TCMSP
1FG9	Interferon-γ	P01579	IFNG	TCMSP
2ERJ	Interleukin-2	P60568	IL2	TCMSP
1IRS	Interleukin-4 receptor subunit α	P24394	IL4R	TCMSP
1Z92	Interleukin-2 receptor subunit α	P01589	IL2RA	TCMSP
5VQP	TGFB1	P01137	TGFB1	TCMSP
2W96	Cyclin-dependent kinase 4	P11802	CDK4	TCMSP
2W99	G1/S-specific cyclin-D1	P24385	CCND1	TCMSP
5E0U	CDKN1A	P38936	CDKN1A	TCMSP
5LAP	Cellular tumor antigen p53	P04637	TP53	TCMSP
5U4K	Transcription factor p65	Q04206	RELA	TCMSP

## Discussion

Apoptosis is a gene-regulated cellular death process, also known as programmed cell death, which occurs in all living cells and is regulated by genes, characterized by a series of cellular morphological changes, including chromosome condensation, nuclear fragmentation, cell shrinkage, the formation of apoptosome, and so on [12]. Two different death signaling pathways are leading to apoptosis: the extrinsic death receptor-dependent pathway and the intrinsic mitochondria-dependent pathway [13,14]. For the intrinsic mitochondria-dependent pathway, the release of cytochrome C from mitochondria into the cytosol is fundamental to apoptosome formation and caspase 3 activations [15]. The members of the Bcl-2 family are vital mediators of cytochrome C release in the context of apoptotic stimuli. Within the Bcl-2 family, Bax is a pro-apoptotic protein and Bcl-2 is an anti-apoptotic protein. Bax or Bcl-2 may control mitochondrial permeability and facilitate the passage of cytochrome C [16]. Thus, the Bax/Bcl-2 ratio determines the fate of many cells [17]. An imbalance of Bax and Bcl-2 proteins may lead to the loss of MMP and the release of cytochrome C, which triggers caspase 3 activation and results in apoptosis [18]. Pro-caspase 3 is cleaved from Asp28-Ser29 and Asp175-Ser176 during activation to form active caspase3 [19]. The most critical substrate for caspase3 is PARP, which is involved in repair and gene integrity monitoring. At the initiation of apoptosis, the PARP of 116 KD was caspase-cut into two fragments of 31 and 85 KD between Asp216 and Gly217, leading to the two zinc finger structures bound to DNA in PARP separated from the catalytic region of the shuttle end and not being able to perform normal functions [20]. As a result, the activity of the Ca^2+^/Mg^2+^ dependent endonuclease affected by the negative regulation is increased, and the DNA between the nucleosomes is cleaved, eventually causing apoptosis.

Cell cycle control is tightly regulated physiologically by CKIs. Members of the Cip family (p21 Cip1) bind to cyclin–CDK complexes and inhibit their activities. Among the transcription targets of p53, p21 Cip1 plays a crucial role in mediating G1 arrest. Besides, the CCND1/CDK4 complex p-Rb on serine residues, thereby canceling Rb’s growth-repressive functions [21], which are caused by Rb’s negative control of a family of heterodimer transcriptional regulators, collectively termed the E2F [22]. Unphosphorylated Rb prevents cell proliferation by binding to and inactivating E2Fs [23]. Phosphorylation of Rb by CDK/cyclin complexes results in the release of active E2F species that stimulate the transcription of genes whose products are necessary for the G1-S transition and S-phase progression.

In our study, we found that Tet could inhibit colon cancer cell growth *in vitro*. Tet could up-regulate the pro-apoptotic gene Bax expression and decreased the anti-apoptotic gene Bcl-2 expression. It disrupted the membrane potential of the mitochondrion and led to the cleavage of caspase 3 and PARP. Colon cancer cells underwent apoptosis after Tet treatment. Moreover, the characteristic of cancer is that cells proliferate uncontrollably [24]. The cell cycle analysis by flow cytometry showed that the percentage of cells in S and G2 phases decreased whereas the G1 phase increased with increasing concentrations of Tet. It suggests that Tet inhibits cell proliferation at an early stage of the cell cycle. The dual-function of Tet led to the dramatic inhibitory effect on colon cancer cells. At the same time, molecular docking results showed that Tet could stably bind to CCND1/CDK4 complex, and its binding mode is similar to its inhibitor. Under the condition of inactivation of p21 protein, CCND1, CDK4, p-Rb protein levels can be down-regulated, and cell proliferation can be inhibited. These results suggest that Tet may represent an effective drug for use in colon cancer therapy.

Meng et al. [25]. found that Tet-induced early G1 arrest is mediated by at least different mechanisms. Tet increases the expression of p53 and p21 Cip1 in wt p53 colon cancer HCT116 cells and induces the proteasome-dependent degradation of CDK4, CDK6, CCND1, and E2F1. CDK6 and CDK4 started to decrease after 2 h of exposure to Tet alone. Pretreatment with MG132 blocked the Tet-induced reduction of CDK6 and CDK4, suggesting the involvement of the 26S proteasome in the degradation of CDK6 and CDK4. Similarly, these experimental results show that CCND1 expression in HT-29 cells is reversed after treated with MG132. We can conclude that the degradation of CCDN1/CDK4 complex is related to the activation of the proteasome pathway, and Tet can activate the proteasome degradation pathway and reversed by MG132. Wu et al. [26] showed that Tet induced significant apoptosis of cultured and subcutaneous mouse colon cancer CT-26 cells. It had substantial effects on tumors, including slower growth and longer animal survival time and higher survival rate. Besides, Singh et al. [27] found that Tet inhibited growth and promoted cell death of pancreatic cancer cells in both dose and time-dependent manner with an IC_50_ in the range of 5–10 μM at 72 h. Xing et al. [28] found that Tet significantly decreased cell proliferation in a dose-dependent manner and induced G1-phase arrest in both MCF-7 and MDA-MB-231 breast cancer cell lines. The mechanism may be reduced expression of CCND1, CCND3, cyclin E, and increased expression of the CKIs, p21/WAF1, and p27/KIP1. Above all, Tet is a promising drug for treating cancer, and for enhancing the efficacy of clinical drugs.

## Funding

This work was supported by Major State Basic Research Development Program (973 Program) of China [grant number 2013CB531801].

## Abbreviations

Bax: Bcl-2 associated X protein
Bcl-2: B-cell lymphoma-2
CCND: cyclin D
CDK: cyclin-dependent kinase
CDKN1A: cyclin-dependent kinase inhibitor 1
E2F1: transcription factor E2F1
FBS: fetal bovine serum
GOL: glycerol
MMP: mitochondrial membrane potential
MTT: methylthiazolyldiphenyl-tetrazolium bromide
NC: nitrocellulose
PARP: poly[ADP-ribose]polymerase 1
PBST: phosphate buffer saline Tween
PI: propidium iodide
pNA: p-nitroaniline
PS: phosphatidylserine
Tet: tetrandrine
TGFB1: transforming growth factor β-1
wt: wild-type
